# Metabolic reprogramming of glutamine is associated with M2 macrophage polarization in allergic rhinitis

**DOI:** 10.3389/fimmu.2026.1815245

**Published:** 2026-05-21

**Authors:** Shang Yan, Kai Xue, Yinan Wang, Desheng Jia, Yongchao Chen, Xueyou Lai, Chunrui Zhao, Guowei Chen, Yang Xu, Xiaoxu Wang, Hongguang Pan, Nan Zeng

**Affiliations:** 1 Department of Otolaryngology, Shenzhen Nanshan People's Hospital, Shenzhen, Guangdong, China; 2 Department of Otolaryngology, Shenzhen Children’s Hospital, Shenzhen, Guangdong, China; 3 ENT Institute and Department of Otolaryngology, Eye & ENT Hospital, Fudan University, Shanghai, China; 4 Department of Obstetrics, Obstetrics and Gynecology Hospital, Fudan University, Shanghai, China

**Keywords:** fibroblast growth factor receptor 1, glutamine, immunometabolism, macrophage polarization, metabolomics, single-cell RNA sequencing

## Abstract

**Introduction:**

Allergic rhinitis (AR) is classically regarded as a type 2 immune-driven disease, yet its chronicity and heterogeneity suggest that additional regulatory layers shape the local immune microenvironment. Although immune cell function is closely linked to metabolic state, how specific metabolic cues are integrated into immune signaling in AR remains unclear.

**Methods:**

We combined human nasal mucosal metabolomic analysis, murine models, single-cell transcriptomics, bulk RNA sequencing, and protein assays to investigate the role of glutamine in AR pathophysiology.

**Results:**

We identified elevated glutamine levels in AR nasal mucosa and found that dietary glutamine supplementation was associated with altered nasal behavioral responses in experimental AR. Single-cell profiling revealed changes in immune cell composition, with macrophages displaying a shift toward an M2-like transcriptional state under high-glutamine conditions. Transcriptomic and pathway analyses positioned fibroblast growth factor receptor 1 (FGFR1) within differentially enriched signaling networks, and its expression increased under high-glutamine conditions. Protein-level assays further showed that aminoacylation-associated signals on FGFR1 varied with glutamine availability, together with coordinated changes in YARS and SIRT1.

**Discussion:**

These hypothesis-generating findings support an associative model in which metabolic alterations in AR are linked to non-canonical modification of FGFR1 and macrophage transcriptional polarization, suggesting a potential immunometabolic layer regulating the nasal mucosal microenvironment.

## 1 Introduction

Allergic rhinitis (AR) is a common chronic inflammatory disorder characterized by nasal obstruction, rhinorrhea, sneezing, and itching. It affects hundreds of millions of individuals worldwide and is closely associated with lower airway diseases such as asthma (1, 2). For decades, research on AR has largely focused on type 2 immune responses centered on T helper 2 (Th2) cells, emphasizing IgE-mediated activation of effector cells and the release of inflammatory mediators in disease pathogenesis (3–5). Current therapeutic strategies based on antihistamines, corticosteroids, and allergen immunotherapy rely heavily on this classical framework (1, 6). However, the chronicity, frequent relapse, and inter-individual variability observed in clinical practice suggest that the pathogenesis of AR may not be determined solely by adaptive immunity abnormalities (7, 8).

In recent years, the emergence of immunometabolism has provided new perspectives for understanding chronic inflammatory diseases (9–11). Accumulating evidence indicates that immune cell activation, differentiation, and functional maintenance critically depend on the availability of metabolic substrates and the configuration of metabolic pathways (12–14). Compared with asthma or inflammatory bowel disease, studies addressing metabolic regulation in AR remain relatively limited, although recent metabolomic analyses and systematic reviews have begun to define AR-associated metabolic signatures (15, 16). How specific metabolic molecules participate in immune regulation in AR remains insufficiently defined.

Glutamine is one of the most abundant non-essential amino acids in the body and serves dual roles in immune cells as both an energy substrate and a regulatory signal (17, 18). In macrophages, metabolic programs are tightly coupled to functional phenotypes (9, 19). Previous studies have suggested that in diseases dominated by type 2 immune responses, glutamine metabolism may be associated with a transcriptional shift of macrophages toward an M2-like state (20, 21). However, within the specific pathological context of AR, whether glutamine shapes the immune microenvironment and its molecular targets remains unclear.

At the level of signal transduction, fibroblast growth factor receptor 1 (FGFR1) is a classical receptor tyrosine kinase governing cell proliferation, differentiation, and metabolic regulation (22, 23). Certain signaling molecules can be finely regulated by non-canonical post-translational modifications, converting intracellular metabolic states directly into signaling outputs (24–26). Aminoacylation represents a recently appreciated class of such modifications, characterized by the transfer of amino acids to lysine residues via aminoacyl-tRNA synthetases, embedding metabolic availability within protein structure (27, 28). Specific, associative connections between such processes and immune receptors in AR-associated signaling have not been fully explored.

Specifically, we examined whether glutamine elevation in AR correlates with remodeling of the immune microenvironment and whether FGFR1 is associated with aminoacylation-related signals under altered glutamine conditions.

## 2 Materials and methods

### 2.1 Transcriptomic and metabolomic analyses of human nasal mucosal samples

#### 2.1.1 Reanalysis of public transcriptomic data

The nasal mucosal transcriptomic dataset GSE206149 was retrieved from the Gene Expression Omnibus (GEO) database (29). This dataset includes samples from 16 AR patients and 19 non-allergic controls. Raw expression matrices were normalized using R software. Differentially expressed genes (DEGs) were identified using FDR-adjusted p-values (p < 0.05 and |log2FC| > 0.5) (30). KEGG pathway enrichment analysis was subsequently performed on the DEGs (31).

#### 2.1.2 Nasal mucosal metabolomics

Nasal mucosal tissues were collected from AR patients and control subjects (5 vs. 5). Following standardized preprocessing, samples were subjected to non-targeted metabolomic profiling. Multivariate statistical analyses were applied to evaluate differences in metabolic profiles between groups (32). Differential metabolites were screened using criteria of VIP > 1, adjusted p < 0.05, and |log2FC| > 0.5. Pathway enrichment analysis was then conducted to map these metabolites within metabolic networks (31).

#### 2.1.3 Quantification of glutamine in nasal mucosa

Glutamine concentrations were quantified in an independent cohort of nasal mucosal samples (AR, n = 8; controls, n = 13) using a commercial colorimetric assay kit (Glutamine Assay Kit (Colorimetric), Abcam/BioVision, ab197011) according to the manufacturer’s instructions. Tissue homogenates were added to a 96-well microplate format, incubated sequentially with detection reagents, developed with chromogenic substrate, and read at 450 nm. Quantification was performed using a standard curve, and samples were measured in replicate wells. Group differences were assessed using two-sided statistical tests.

### 2.2 Animal models and behavioral assessment

Specific pathogen-free (SPF) male C57BL/6 mice were randomly assigned to four groups (10 mice per group). Male mice were selected at the design stage to reduce hormonal and metabolic variability associated with estrous cycling in this initial proof-of-concept model focused on glutamine-associated metabolic changes. However, because allergic rhinitis affects both sexes, this choice limits the generalizability of the *in vivo* findings and should be addressed in future studies including female mice. The group size of 10 animals was predetermined for this exploratory *in vivo* study based on the expected biological variability of OVA-induced behavioral readouts and the possibility of inter-animal heterogeneity in sensitization responses, with the aim of retaining sufficient evaluable animals for behavioral and downstream tissue-based analyses.

Control group: standard diet (glutamine 0.5%), saline sensitization and challenge.
AR group: standard diet (glutamine 0.5%), ovalbumin (OVA) sensitization and challenge.
AR + Low-Gln group: low-glutamine diet (approximately 0%), OVA sensitization and challenge.
AR + High-Gln group: high-glutamine diet (30%), OVA sensitization and challenge.

A 30% glutamine diet constitutes an intentionally extreme, hyper-physiological perturbation model used to increase glutamine-associated metabolic pressure and facilitate exploratory pathway-oriented interrogation *in vivo*, rather than to simulate a clinically relevant dietary supplementation regimen. All invasive procedures were performed under isoflurane anesthesia. The OVA sensitization and intranasal challenge protocol was established as per standard methods for AR models (33). Ten minutes after the final challenge, sneezing and nasal scratching episodes were recorded.

### 2.3 Single-cell RNA sequencing and immune cell subset analysis

Twenty-four hours after behavioral assessment, two biological replicates per group (total n = 8 mice) were collected. This exploratory sample size (n = 2 per group) was used for initial cellular mapping and descriptive comparison of broad subset redistributions, rather than as a stand-alone basis for robust inferential statistics. Fresh nasal mucosal tissues were dissected immediately after collection, minced into small fragments, and enzymatically digested using collagenase and DNase for 45 minutes at 37 degrees C. Single-cell suspensions were filtered through 70-μm cell strainers and maintained briefly at 4 degrees C before library preparation.

Single-cell RNA sequencing (scRNA-seq) was performed utilizing the 10x Genomics Chromium platform (10x Genomics, USA) with Single Cell 3’ chemistry. Sequencing was conducted on an Illumina NovaSeq 6000 system (150-bp paired-end reads). Dimensionality reduction, clustering, and cell annotation were performed using the Seurat package (34), identifying macrophages, T cells, B cells, dendritic cells, neutrophils, and monocytes. Importantly, our macrophage classification (M1 versus M2) is based exclusively on transcriptomic signatures (gene set-based scoring) rather than functional validation (e.g., flow cytometry).

### 2.4 RNA sequencing and enrichment analysis in the *in vitro* model

THP-1 cells were cultured under standard conditions (RPMI 1640 supplemented with 10% fetal bovine serum, 1% penicillin-streptomycin, and standard 2 mM L-glutamine concentration) in a humidified 37 degrees C, 5% CO2 incubator (Control, n = 3) or treated with high-glutamine medium containing 4 mM L-glutamine (n = 3) before harvest for total RNA extraction and transcriptomic sequencing. THP-1 differentiation and IL-4-directed polarization were performed following established THP-1 macrophage differentiation workflows reported previously (35, 36), with PMA pretreatment (100 ng/mL, 24 h) followed by IL-4 stimulation (20 ng/mL) for M2-oriented polarization before downstream glutamine-related treatment and harvest.

Differential expression analysis was performed using DESeq2 (FDR-adjusted p < 0.05 and |log2FC| > 1). GO, KEGG, and Reactome enrichment analyses were subsequently conducted (31, 37).

### 2.5 Western blotting and immunoprecipitation

#### 2.5.1 Tissue and cell sample preparation

THP-1 cells were pretreated with PMA (100 ng/mL, 24 h) and subsequently stimulated with IL-4 (20 ng/mL) to induce M2-oriented differentiation. Cells were then treated with glutamine, the FGFR1 inhibitor PD173074, or the SIRT1 inhibitor EX-527 in the respective experiments, and harvested after 48 h.

#### 2.5.2 Western blotting

Total proteins were extracted using RIPA lysis buffer containing protease and phosphatase inhibitors, and concentrations were determined via BCA assay. Equal amounts of protein (25 μg per lane) were subjected to 10% SDS-PAGE and transferred onto PVDF membranes. Primary antibodies against FGFR1, YARS, and SIRT1 were used overnight at 4 degrees C at 1:1000 dilution. Beta-actin or actin was used as the loading control in the corresponding immunoblots. Signals were detected using HRP-conjugated anti-rabbit IgG secondary antibodies (1:5000). Band intensities were visualized using standard chemiluminescence, and semi-quantitative densitometric analyses were performed in ImageJ to compare relative band intensities across treatment groups.

#### 2.5.3 Immunoprecipitation

Protein lysates (1 mg) were pre-cleared with Protein A/G agarose beads, followed by overnight incubation at 4 degrees C with the primary anti-FGFR1 antibody. Immunocomplexes were precipitated with Protein A/G agarose beads for 2 h at 4 degrees C. Beads were washed extensively and proteins were eluted in SDS sample buffer for downstream Western blotting to assess aminoacylation-associated signals (protein modifications mapped to the precipitated FGFR1 complex).

### 2.6 Statistical analysis

Data distribution normality was specifically assessed using the Shapiro-Wilk test, and variance homogeneity was evaluated with Levene’s test. Continuous variables matching parametric test assumptions are presented as mean +/- SEM and analyzed using two-sided unpaired Student’s t-tests (for two groups) or one-way ANOVA with Tukey’s *post-hoc* tests (for multiple groups). Features violating parametric assumptions were analyzed via the Mann-Whitney U test. For transcriptomic and omics analyses, p-values were corrected using the False Discovery Rate (FDR) approach. Statistical significance was designated as p < 0.05 (or FDR < 0.05).

## 3 Results

### 3.1 Transcriptomic and metabolomic profiling identifies glutamine as a prominently elevated metabolite in allergic rhinitis nasal mucosa

**Figure 1:**
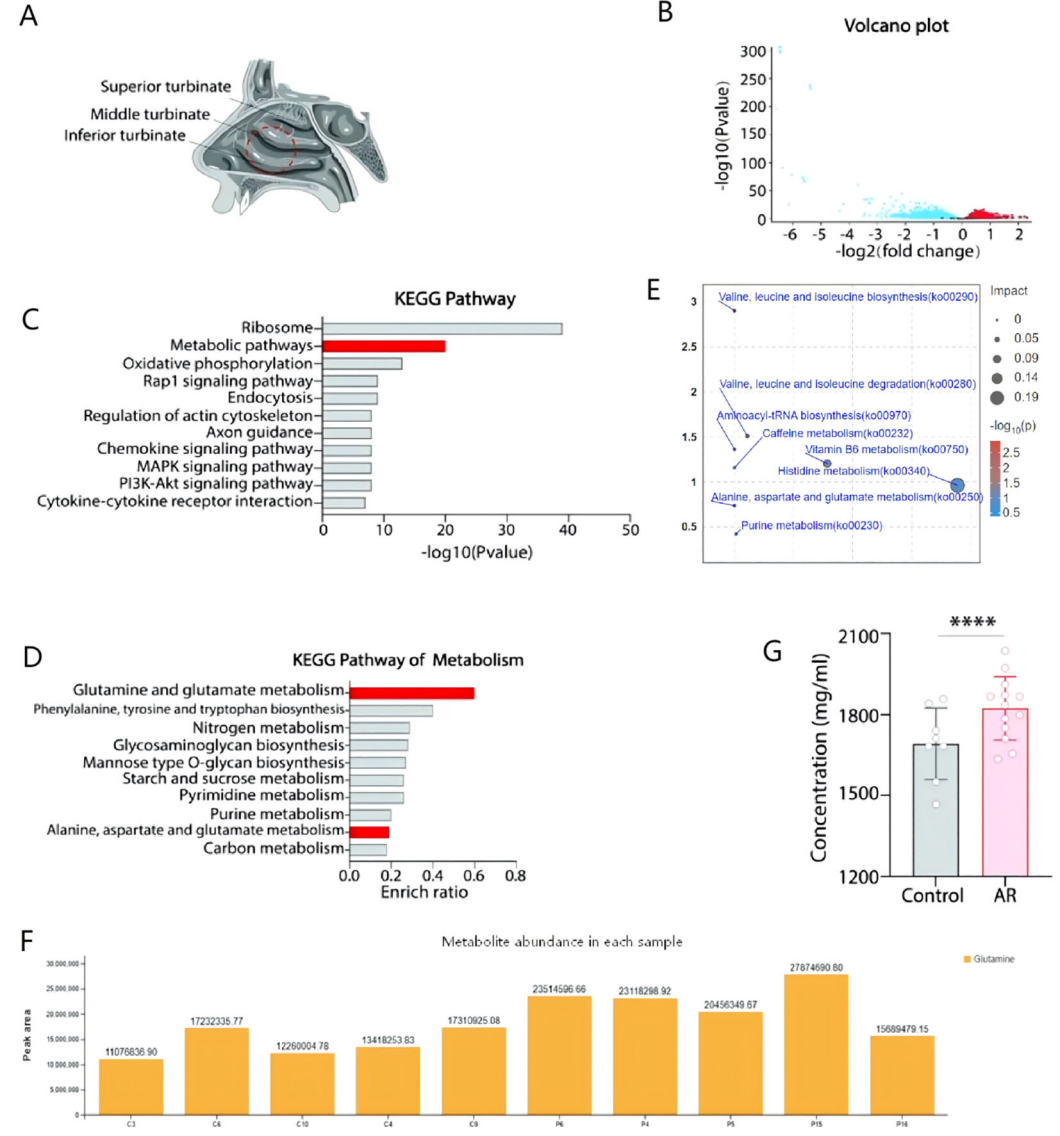
**(A)** Schematic illustration of nasal mucosal sampling sites in the GSE206149 dataset. **(B)** Volcano plot of differentially expressed genes between allergic rhinitis (AR) and control nasal mucosa. **(C)** KEGG pathway enrichment analysis of 5,244 differentially expressed genes (DEGs); red bars highlight enrichment in metabolic pathways. **(D)** Secondary KEGG pathway analysis of 425 metabolism-associated DEGs; red bars indicate pathways related to glutamine metabolism. **(E)** KEGG pathway enrichment of differentially abundant metabolites identified by non-targeted metabolomics. **(F)** Relative abundance of glutamine in individual nasal mucosal samples based on metabolomic peak areas. **(G)** Quantification of glutamine concentration in nasal mucosal tissues from control and AR subjects using a colorimetric assay (**** p < 0.0001).

To systematically characterize molecular alterations in the nasal mucosa of allergic rhinitis (AR), we first reanalyzed transcriptomic data from the public dataset GSE206149. This dataset includes nasal brushing samples from 16 AR patients and 19 non-allergic controls, with sampling locations illustrated in **Figure 1A**. Differentially expressed genes (DEGs) were identified using thresholds of FDR-adjusted p < 0.05 and |log2FC| > 0.5, yielding a total of 5,244 DEGs (**Figure 1B**).

KEGG pathway enrichment analysis of these DEGs showed that the most enriched category was the ribosome-related pathway. In addition, “metabolic pathways” emerged as another highly enriched category (highlighted in red), comprising 425 DEGs (**Figure 1C**). Classical signaling pathways, including PI3K and MAPK, were also represented among the enriched terms. To further delineate metabolism-related alterations in AR, these 425 metabolism-associated DEGs were subjected to a second round of KEGG analysis. Among the top 10 enriched metabolic pathways, two were directly related to glutamine metabolism (**Figure 1D**, red bars).

Based on these transcriptomic findings, non-targeted metabolomic profiling was performed on nasal mucosal tissues from AR patients and controls (5 vs. 5). KEGG pathway enrichment analysis of differentially abundant metabolites revealed that altered metabolites were mainly distributed in pathways involving branched-chain amino acids (leucine, isoleucine, and valine), glyoxylate, and glutamine metabolism (**Figure 1E**). Notably, glutamine-related pathways were the only metabolic pathways concurrently enriched in both transcriptomic and metabolomic analyses.

Examination of the maximal peak area of glutamine in the metabolomic dataset, reflecting its relative abundance in tissue samples, demonstrated that glutamine levels were higher in AR nasal mucosa than in controls (**Figure 1F**). To validate these observations, glutamine concentrations were quantified in an independent cohort of nasal mucosal samples using a colorimetric assay kit (AR, n = 8; controls, n = 13). Consistent with the metabolomic results, glutamine levels in AR nasal mucosa were significantly higher than those in non-allergic controls, reaching a high level of statistical significance (**** p < 0.0001) (**Figure 1G**).

Together, these multi-omics and quantitative data indicate that glutamine is consistently elevated in AR nasal mucosa and represents a prominently altered metabolite within the metabolic landscape of allergic rhinitis.

### 3.2 Dietary glutamine supplementation is associated with altered nasal behavioral responses in an experimental model of allergic rhinitis

To assess the functional consequences of altered glutamine availability at the organismal level, nasal-related behavioral responses were systematically evaluated in mice subjected to different dietary conditions following establishment of an allergic rhinitis (AR) model. Mice were assigned to four groups: Control, AR, AR + Low-Gln, and AR + High-Gln. Animals received a standard diet (glutamine 0.5%), a high-glutamine diet (30%), or a low-glutamine diet (approximately 0%) for 2 months. AR was then induced in the three experimental groups using a classical ovalbumin (OVA) sensitization and challenge protocol. During the 10-min period following the final OVA challenge, the numbers of sneezing and nasal scratching episodes were recorded for each mouse. To maintain consistency with subsequent molecular and single-cell analyses, the present section focuses on the behavioral comparison between the AR group maintained on a standard diet and the AR + High-Gln group.

**Figure 2:**
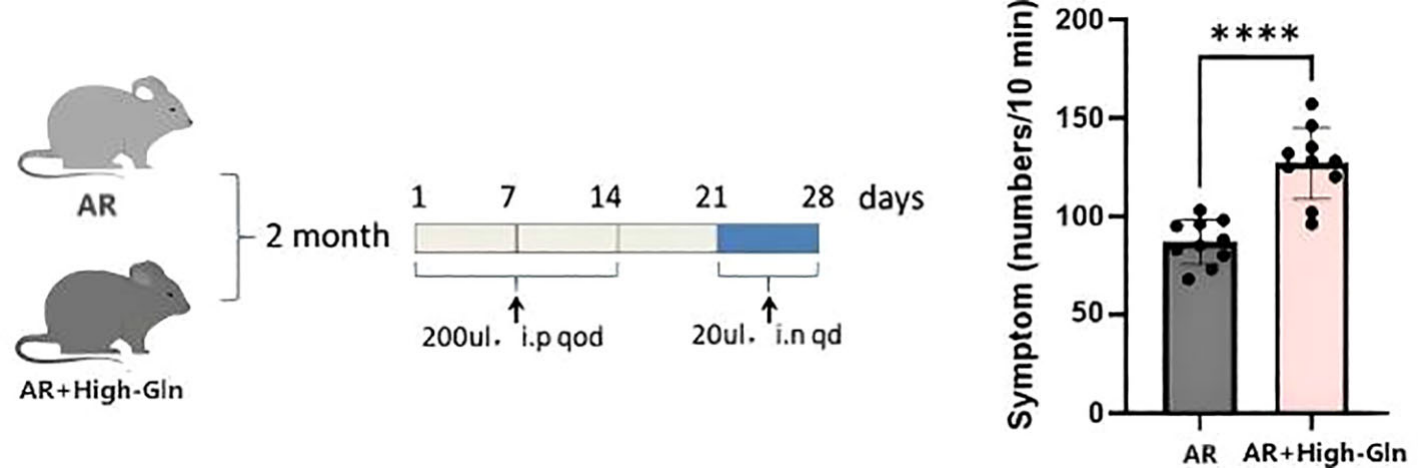
Experimental design of dietary glutamine intervention and ovalbumin (OVA) sensitization in C57BL/6 mice, and quantification of nasal-related behaviors. Mice were maintained on standard or high-glutamine diets for 2 months and subsequently subjected to OVA sensitization and intranasal challenge. Ten minutes after the final OVA challenge, the numbers of sneezing and nasal scratching episodes were recorded as behavioral readouts. Bar plots show the total counts of nasal-related behaviors within a 10-min observation window (**** p < 0.0001).

Following OVA challenge, mice in the AR group receiving the standard diet exhibited approximately 87 sneezing and nasal scratching events within the 10-min observation window. In contrast, mice maintained on the high-glutamine diet for 2 months displayed approximately 127 events under the same stimulation conditions. Both sneezing and nasal scratching counts differed significantly between the two groups, reaching a high level of statistical significance (**Figure 2**) (**** p < 0.0001).

Thus, under identical sensitization and challenge conditions, mice functionally exposed to a hyper-physiological high-glutamine diet exhibited higher counts of both sneezing and nasal scratching than animals receiving a standard diet.

### 3.3 Single-cell profiling reveals a glutamine-associated shift in immune cell composition with preferential M2-like macrophage transcriptional orientation

**Figure 3:**
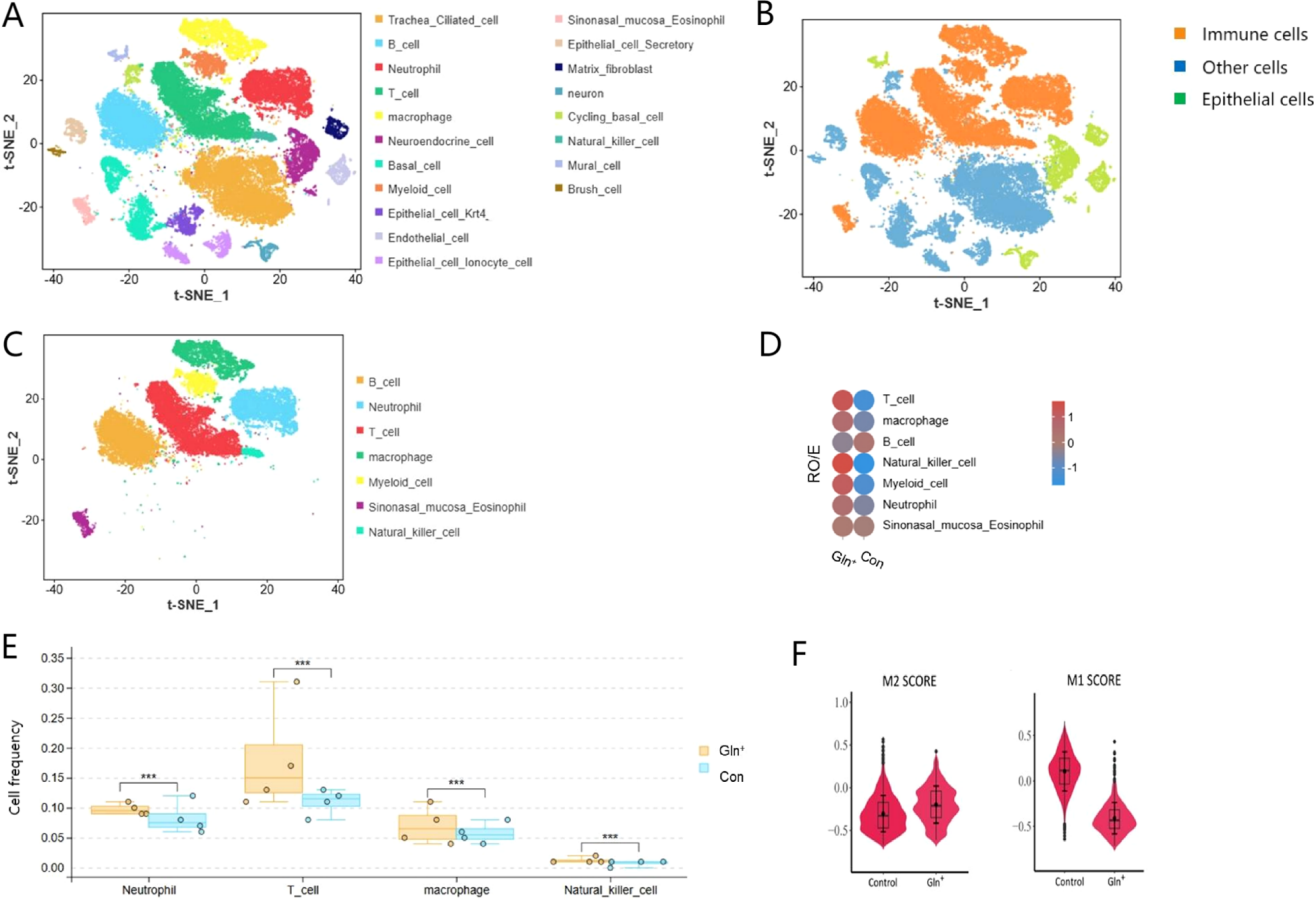
**(A)** t-SNE visualization of 19 cell populations identified from nasal mucosal single-cell RNA sequencing. **(B)** t-SNE plot showing three major cell categories after hierarchical grouping. **(C)** t-SNE visualization of immune cell subsets. **(D)** Relative representation of immune cell subsets across conditions, expressed as observed-to-expected (O/E) ratios. **(E)** Comparison of cell frequencies of major immune subsets between groups; each point represents one biological replicate (*** p < 0.001). **(F)** Distribution of M2 and M1 gene signature scores in macrophage populations under control and high-glutamine conditions.

To delineate the relationship between glutamine availability and immune cell characteristics in the nasal mucosa, single-cell RNA sequencing (scRNA-seq) was performed on nasal mucosal tissues from mice subjected to different experimental conditions. After quality control and data integration, high-quality single-cell transcriptomic maps were obtained, revealing well-defined cellular distributions at the global level. Based on canonical marker genes, multiple immune cell populations were annotated, including macrophages, T cells, B cells, dendritic cells, and granulocytes (**Figures 3A, B**). Distinct differences in immune cell composition were observed across treatment groups, indicating that immune cell proportions varied in a quantifiable manner under different glutamine conditions.

Among all immune cell types, macrophages displayed the most pronounced changes. Further subdivision and comparison of macrophage subpopulations revealed marked heterogeneity in their internal composition across treatment conditions (**Figures 3C–E**). Under high-glutamine conditions, the relative proportion of macrophage subsets transcriptionally associated with alternative activation increased, whereas the proportion of subsets associated with classical activation decreased, reflecting a structural redistribution within the macrophage compartment.

To assess trends in macrophage transcriptional states in a standardized manner, M1 and M2 gene signature scoring was performed at the single-cell level using a simplified M1/M2-oriented transcriptional framework informed by recent macrophage heterogeneity literature (38). Under high-glutamine conditions, macrophages exhibited an overall increase in M2-associated scores accompanied by a relative decrease in M1-associated scores (**Figure 3F**). Importantly, our macrophage classification is based firmly on transcriptomic signatures (gene set-based scoring) rather than functional validation, and no parallel flow cytometry, cytokine profiling, or macrophage functional assays were performed in this study. Accordingly, the classic M1/M2 framework is used here only as an oversimplified transcriptional reference for macrophage-state trends rather than as a definitive functional classification.

### 3.4 RNA-seq profiling identifies differential gene expression and pathway enrichment associated with high-glutamine treatment, highlighting FGFR1 within enriched signaling contexts

**Figure 4:**
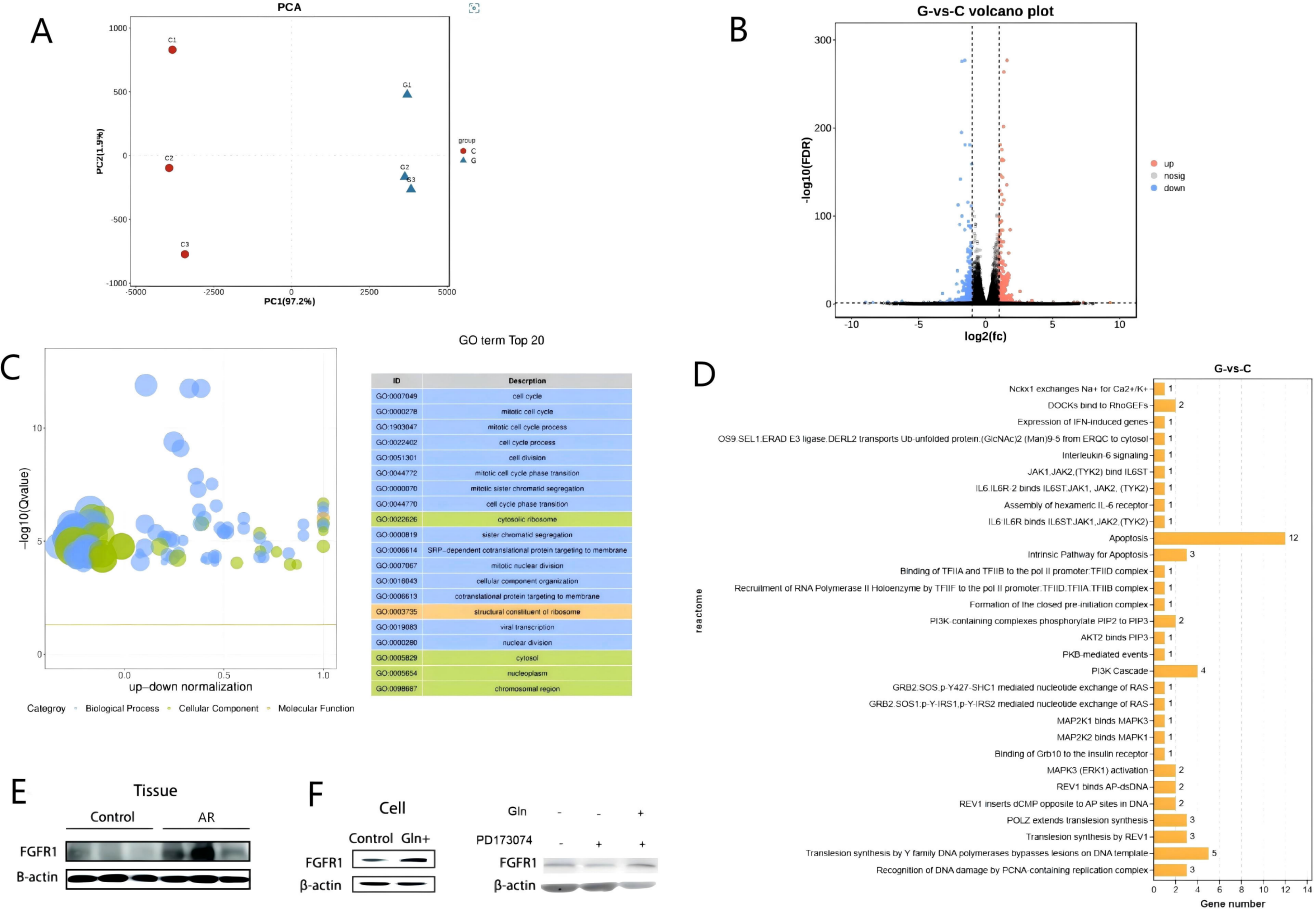
**(A)** Principal component analysis (PCA) of RNA-seq profiles from THP-1 cells under control and high-glutamine conditions. **(B)** Volcano plot of RNA-seq data. Differential expression thresholds were set at FDR-adjusted p < 0.05 and |log2FC| > 1. **(C)** Gene Ontology (GO) enrichment analysis of differentially expressed genes. **(D)** Reactome pathway enrichment analysis of differentially expressed genes. **(E)** FGFR1 protein expression in normal nasal mucosa and allergic rhinitis nasal mucosa. **(F)** FGFR1 protein expression in THP-1 cells under control conditions, after glutamine supplementation, after PD173074 treatment, and after combined glutamine and PD173074 treatment.

To characterize transcriptomic alterations under high-glutamine conditions, RNA sequencing was performed on THP-1 cells cultured under standard conditions (Control, n = 3) and high-glutamine treatment (High-glutamine, n = 3). Principal component analysis (PCA) based on the gene expression matrix revealed distinct clustering of the two groups. Samples within each group were closely distributed, whereas the two groups were clearly separated, indicating that overall transcriptional profiles differed between treatment conditions (**Figure 4A**).

Differential expression analysis was conducted using DESeq2 with thresholds of FDR-adjusted p < 0.05 and |log2FC| > 1. A total of 646 differentially expressed genes (DEGs) were identified, including 226 upregulated and 420 downregulated genes (**Figure 4B**). These DEGs exhibited a bilateral distribution on the volcano plot, reflecting discrete transcriptional differences between the two groups.

Functional enrichment analysis was subsequently performed using the DEG set. Gene Ontology (GO) analysis indicated that DEGs were primarily enriched in categories related to Biological Process (cell cycle, cell division) and Cellular Component (cytoplasm, nucleus, chromatin region) (**Figure 4C**).

At the pathway level, Reactome-based enrichment analysis showed that DEGs were mainly distributed across apoptosis-related pathways, the PI3K signaling pathway, the MAPK signaling pathway, and DNA damage-related pathways (**Figure 4D**).

Within this enrichment context, FGFR1 appeared among the gene sets associated with multiple differentially enriched pathways. Consistent with the transcriptomic analysis, fibroblast growth factor receptor 1 (FGFR1) displayed an upregulated trend under high-glutamine conditions. At the protein level, FGFR1 expression was higher in allergic rhinitis tissues than in control tissues (**Figure 4E**). In the *in vitro* model, FGFR1 expression in THP-1 cells increased under high-glutamine conditions relative to control conditions; lower expression was observed in the PD173074-treated group, whereas the combined PD173074 plus glutamine group showed higher FGFR1 levels than the PD173074-only group (**Figure 4F**). All comparisons reached statistical significance.

### 3.5 FGFR1 aminoacylation-associated signals vary with glutamine availability, mapping to YARS- and SIRT1-dependent contexts

**Figure 5:**
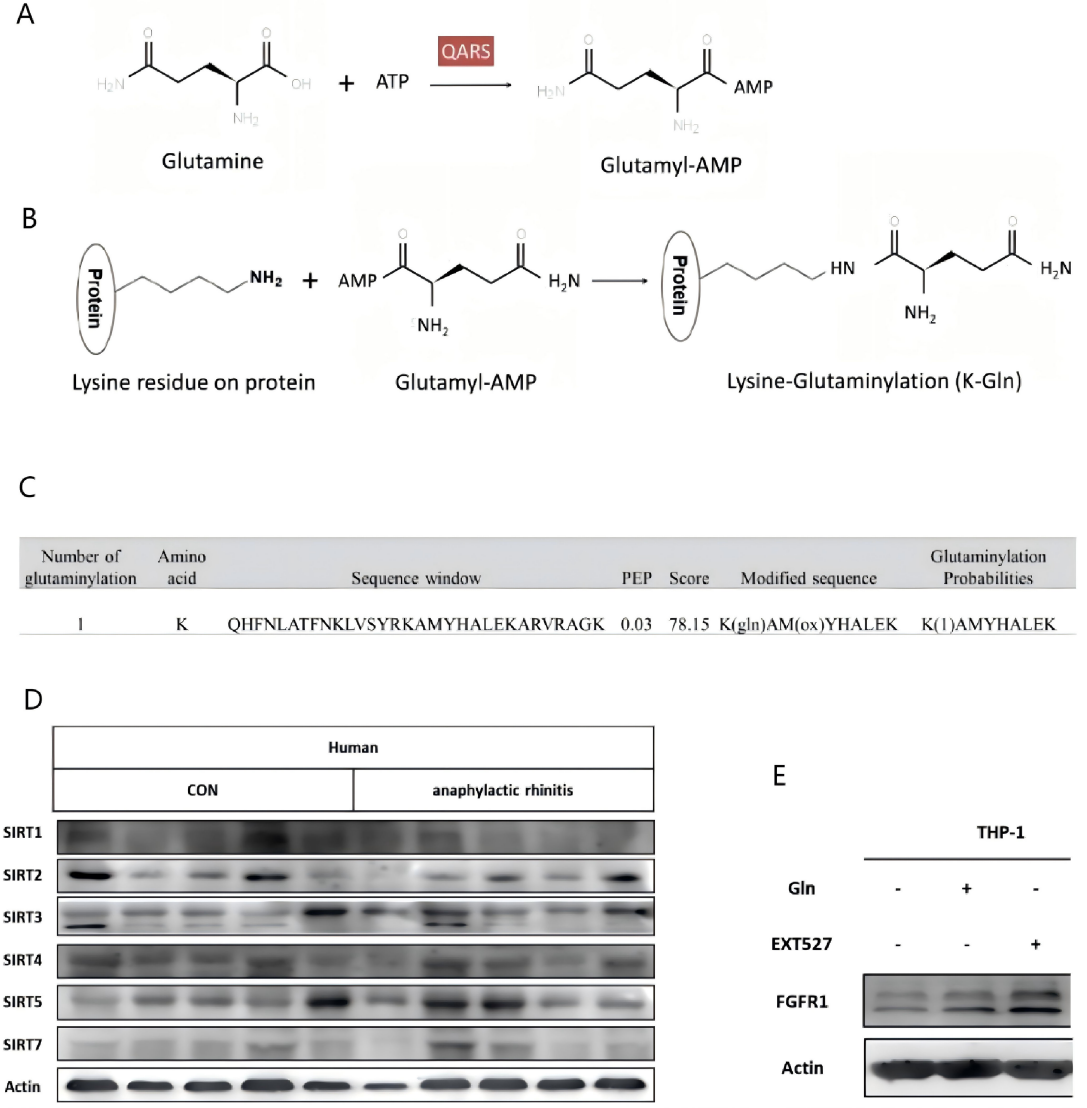
**(A, B)** Schematic illustration of protein aminoacylation mediated by glutamine, showing the formation of glutamyl-AMP and subsequent lysine glutamylation. **(C)** Predicted glutamine-dependent aminoacylation sites of FGFR1 based on the aminoacylation modification database. **(D)** Expression of SIRT family proteins in normal nasal mucosa and allergic rhinitis tissues. **(E)** FGFR1 protein expression in THP-1 cells under control conditions and after separate treatment with glutamine or EX-527.

To provide conceptual context for the protein analyses, **Figure 5A** and **5B** illustrate the aminoacylation-related framework examined in this study, and **Figure 5C** summarizes putative glutamine-dependent lysine-glutamine (K-Gln) modification sites predicted on FGFR1 using aminoacylation modification databases.

YARS and SIRT1 were selected as biologically relevant candidate regulators within our aminoacylation- and metabolism-oriented framework. YARS was examined because aminoacyl-tRNA synthetases are directly linked to amino acid utilization and therefore provide a plausible biochemical context for aminoacylation-associated signals, whereas SIRT-family proteins are well-recognized regulators of metabolic adaptation and protein modification states under nutrient stress. Within the same experimental system, YARS expression was examined in parallel. As YARS protein levels elevated under high-glutamine conditions, they were accompanied by increased FGFR1 aminoacylation-associated signals within the precipitated complex, highlighting an associative covariance among these factors. When aminoacylation-related processes were experimentally perturbed, the magnitude of these changes was reduced. These parameters differed quantitatively among treatment groups and reached statistical significance.

The effects of SIRT1-related modulation were then examined in sequence. At the tissue level, expression of SIRT family proteins was assessed, revealing that SIRT1 protein levels were lower in AR tissues than in control tissues (**Figure 5D**). *In vitro*, THP-1 cells differentiated with PMA and IL-4 were treated separately with glutamine or the SIRT1 inhibitor EX-527. After 48 h, FGFR1 protein levels were analyzed. Both glutamine treatment and EX-527 treatment were associated with increased FGFR1 expression relative to control conditions (**Figure 5E**). Differences among groups reached statistical significance.

## 4 Discussion

This study integrates human nasal mucosal samples, animal models, single-cell transcriptomics, and bulk RNA sequencing to conceptually navigate the axis of “glutamine-FGFR1 expression-macrophage polarization” as an associative landscape extending beyond pure Th2 classical immune frameworks. We observed elevated glutamine levels in human AR nasal mucosa; concurrently, extreme high-glutamine experimental conditions in mice correlated with an increase in M2-like macrophage transcriptomic profiles. At the molecular level, FGFR1 was identified within differentially enriched analyses, with its expression varying alongside YARS and SIRT1, two candidate regulators selected because of their biological relevance to aminoacylation-associated and metabolic regulatory processes. These correlations compose a hypothesis-generating scaffold linking metabolically rich environments to adaptive signaling nuances.

Advances in immunometabolism increasingly assert that metabolic substrates and pathways direct immune cell behavior as forcefully as cytokine networks (14, 39). In this context, our study suggests an associative intersection between metabolic status and non-canonical regulation of FGFR1. Rather than demonstrating direct biochemical modification, the present data support a hypothesis-generating framework in which metabolic abundance may be linked to FGFR1-associated aminoacylation signals and related immune changes (24–26, 40–42).

Our single-cell analyses reveal quantitative transcriptomic variability within macrophage subset distributions under high-glutamine pressure. Under high-glutamine conditions, macrophage transcriptomic tendencies gravitate toward an M2-like state. Because macrophages possess extreme phenotypic plasticity dynamically linked with metabolic health (38, 39), they frequently act as cellular interfaces linking systemic metabolic signals to localized immunity shifts, likely reinforcing the broader type 2 immunity environment seen in AR.

Several major limitations of this work warrant fundamental emphasis. First, the central relationship tying glutamine directly to FGFR1 aminoacylation-associated signals and macrophage reprogramming is purely associative. We did not perform structure-specific lysine mutagenesis, rescue experiments, or definitive biochemical validation such as LC-MS/MS; future studies will be required to determine whether FGFR1 aminoacylation is structurally present and functionally relevant *in vivo*. Second, the current *in vivo* evidence is weighted toward glutamine supplementation and does not determine whether glutamine restriction can reverse or normalize AR-related immune and behavioral features at the organismal level; this question should be addressed in future dedicated intervention studies. Third, the small human sample sizes used for metabolomics (n = 5 vs 5) and colorimetric glutamine quantification (n = 8 vs 13) limit statistical stability and increase susceptibility to sampling bias, and these human observations should therefore be interpreted as exploratory rather than definitive. Fourth, our *in vivo* experiments were conducted only in male mice, a design choice made to reduce hormonal variability in an initial proof-of-concept metabolic study; however, this restricts extrapolation across sexes, and future studies should include female animals to determine whether the observed glutamine-associated immune and behavioral changes are sex dependent. Fifth, our macrophage subtype profiling fundamentally relies on simplified bioinformatics scoring; our macrophage classification is based exclusively on transcriptomic signatures without parallel functional validation (e.g., via CD206 or iNOS flow cytometry assays) recognizing the constraints of forcing complex continuous macrophage phenotypes into a binary M1/M2 categorization. Sixth, the deployment of a 30% glutamine diet constitutes an intentionally extreme, non-physiological perturbation and therefore has limited translational relevance to human dietary exposure; future studies using graded and more physiologically relevant glutamine interventions will be necessary to clarify dose dependence and off-target metabolic effects. Seventh, no spatial transcriptomic approaches or histological validation methods (such as IHC or immunofluorescence) were applied in this study; consequently, we could not determine the tissue-level localization of macrophage subsets, the structural distribution of FGFR1-related signals, or their spatial relationships within the nasal mucosal architecture.

Overall, AR pathogenesis spans adaptive immune dysfunction through environmental and complex metabolic variables. Glutamine’s putative role highlights potential non-canonical protein interplay providing broader interpretations of how chronic immune signaling structures interpret surrounding metabolic nutrient cues.

## 5 Conclusion

This study shows that elevated glutamine levels in allergic rhinitis nasal mucosa are associated with remodeling of the local immune microenvironment and with a transcriptomic shift of macrophages toward an M2-like state. At the molecular level, these changes are accompanied by altered FGFR1 expression and by variation in aminoacylation-associated signals together with YARS and SIRT1. These findings are associative and hypothesis-generating, and they support further investigation into how metabolic cues may shape immune signaling in allergic rhinitis.

## Data Availability

The public transcriptomic dataset reanalyzed in this study is available in the Gene Expression Omnibus under accession number GSE206149. Other data generated or analyzed during this study are included in this article, and additional supporting materials are available from the corresponding authors on reasonable request.
